# Enamel Remineralizing Agents: State of the Art

**DOI:** 10.3390/ma19122550

**Published:** 2026-06-12

**Authors:** Elizabeta Gjorgievska, Marija Stevanovic, Aleksandar Dimkov, John W. Nicholson

**Affiliations:** 1 Department of Pediatric and Preventive Dentistry, Faculty of Dentistry, Ss Cyril and Methodius University in Skopje, 1000 Skopje, North Macedonia; mstevanovic@stomfak.ukim.edu.mk (M.S.); adimkov@stomfak.ukim.edu.mk (A.D.); 2 Bluefield Centre for Biomaterials Ltd., London EC1V 2NX, UK; jwnicholson01@gmail.com

**Keywords:** remineralization, demineralization, enamel, dental caries, calcium phosphate, fluoride, biomimetic remineralization, casein phosphopeptide, hydroxyapatite, bioactive glass

## Abstract

Dental caries remains the most prevalent chronic disease worldwide, yet early enamel lesions are reversible if managed with appropriate remineralizing agents. This narrative review synthesizes current evidence on remineralizing agents, their mechanisms of action, and clinical applications, with a focus on dental materials used in preventive and minimally invasive dentistry. Traditional fluoride-based approaches enhance remineralization through fluorapatite formation; however, their effectiveness is limited when calcium and phosphate bioavailability is insufficient. Biomimetic agents, including casein phosphopeptide–amorphous calcium phosphate (CPP-ACP), bioactive glasses, tricalcium phosphate, and nano-hydroxyapatite, provide these bioavailable ions and demonstrate superior performance under challenging clinical conditions. Emerging therapies such as probiotics, photodynamic therapy, and laser-assisted mineralization show promise but require further clinical validation. Based on the primary mechanism of action, an original classification of remineralizing agents is proposed, grouping them into fluoride-based agents, calcium-phosphate systems, nanotechnology-based systems, biofilm modifiers, biomimetic and emerging systems, and adjunctive antimicrobial therapies. The review concludes that bioavailable calcium represents a critical limiting factor in remineralization under certain conditions, and that combination protocols incorporating multiple remineralizing agents, tailored to individual patient risk profiles, achieve superior outcomes compared to single-agent approaches. Clinicians are encouraged to adopt minimally invasive, patient-tailored remineralization strategies that arrest lesions before cavitation, preserving natural tooth structure and reducing the lifelong restorative burden.

## 1. Introduction

Demineralization and remineralization are dynamic processes characterized by the exchange of calcium and phosphate between saliva and hard dental tissues. Therefore, to prevent the progression of demineralization, it is necessary to balance these processes [1,2].

The chemical basis of these processes is similar in enamel, dentin, and root cementum. However, differences in structure and the relative amounts of minerals and organic matter in these tissues result in significant differences in the nature and progression of the lesion [3,4].

**Figure 1:**
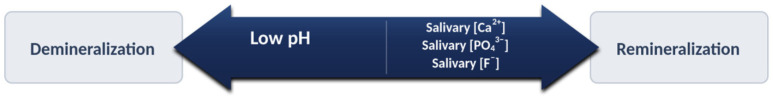
Enamel de/remineralization cycles.

The balance between demineralization and remineralization depends on several factors present in saliva and plaque, such as the concentrations of salivary calcium and phosphate, pH value, and the bioavailability of fluorides (Figure 1). At low pH values, dissolution of calcium and phosphate occurs in the hydroxyapatite (HAp) crystal structure of the tooth (demineralization). In the initial phases, this dissolution can be reversed by remineralization from available calcium and phosphate in saliva. Hence, we are dealing with cyclical changes in the oral environment that result in alternating periods of demineralization and remineralization at the interface between the tooth and the oral medium [5,6].

Demineralization occurs as a process during tooth decay and can also be observed following tooth whitening/bleaching, as well as in hypersensitive teeth [7,8]. Therefore, indications for stimulation of remineralization include the following: treatment of initial forms of dental caries; additional preventive therapy for caries reduction in high-risk patients; before and after tooth whitening/bleaching; and desensitization of sensitive teeth [9,10].

Recent studies have provided updated evidence on caries etiology, and also advanced antimicrobial and remineralizing strategies [11,12]. This review integrates the most recent scientific data with the current clinical protocols. Given the rapid expansion of remineralizing technologies and the persistent global burden of dental caries, this review aims to synthesize the mechanisms of action and clinical evidence for currently available remineralizing agents, propose an evidence-based classification, and provide clinically oriented guidance for their application in preventive and minimally invasive dentistry.

## 2. Methods/Search Strategy

This narrative review is based on a literature analysis conducted using ScienceDirect and PubMed, supplemented with additional references identified through citation tracking. The keywords used were as follows: remineralization; demineralization; enamel; dental caries; calcium phosphate; fluoride; biomimetic remineralization; casein phosphopeptide; hydroxyapatite; bioactive glass.

The searches covered the period 2010–2025 and identified 73 references published during this time period. The review was further augmented using foundational older references cited in these papers, as well as papers already known to the authors. In this way, relevant literature spanning more than five decades has been identified and incorporated into the article.

**Scheme 1:**
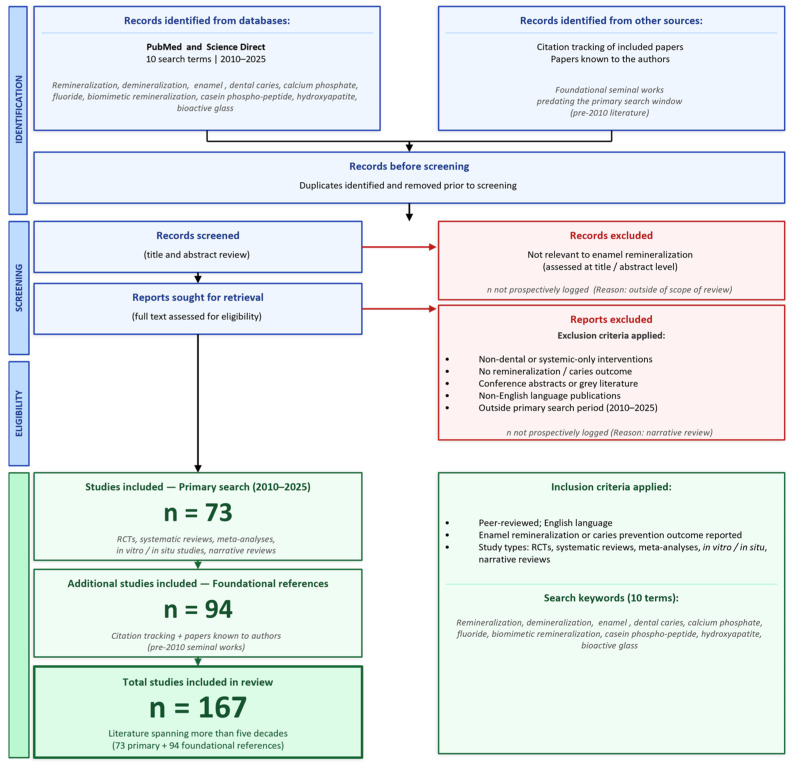
Preferred Reporting Items for Systematic Reviews and Meta-Analyses (PRISMA) flow diagram of the literature search (adapted for a narrative review).

Inclusion criteria comprised peer-reviewed publications in English reporting on remineralizing agents for dental enamel, including randomized controlled trials, systematic reviews, meta-analyses, in vitro and in situ studies, and narrative reviews. Studies were included if they reported outcomes related to enamel remineralization, demineralization inhibition, or caries prevention. Studies on non-dental applications or purely systemic interventions without relevance to enamel remineralization were excluded, as were conference abstracts and gray literature. Given the heterogeneity of study designs, a narrative synthesis was adopted. The quality of included evidence was assessed qualitatively with attention to study design, sample size, and whether outcomes were derived from in vitro, in situ, or clinical settings; levels of evidence are indicated throughout the text. The PRISMA flow diagram adapted for a narrative review shows the studies evaluated in the review (Scheme 1).

The clinical significance of enamel remineralization has thereby been described in detail, the main remineralizing agents systematically reviewed, and the main mechanisms of action discussed in the context of current knowledge on de/remineralization dynamics in the oral environment.

## 3. De/Remineralization Processes

Enamel is a complex, avital, secretory product of the specialized epithelial cells–ameloblasts [13]. In mature enamel, HAp crystals are approximately 30–40 nm wide and ~10 μm long, while the diameter of enamel rods is about 4–6 μm. It consists of ≈96 wt% HAp and ≈4 wt% organic material (non-collagen protein, including enamelins and residual matrix proteins) and water [13,14]. HAp in enamel has a hexagonal form and contains a greater amount of minerals compared to dentin and cementum [13,14].

**Figure 2:**
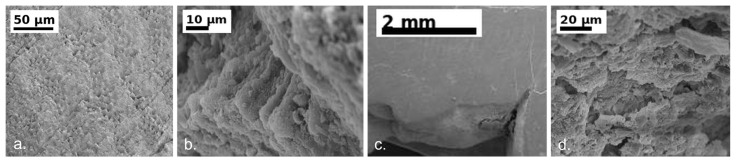
Enamel demineralization (scanning electron micrographs; SEM). Demineralization initially leads to an increase in porosity in the interprismatic spaces, and later in the prisms themselves: (**a**) honeycomb appearance on the enamel surface, resembling enamel etched with orthophosphoric acid before application of a composite restoration; (**b**) increase in the volume of the interprismatic spaces, with a decrease in the diameter of the enamel prisms; (**c**,**d**) with further demineralization, there is a complete loss of the prismatic structure of the enamel.

The demineralization process refers to the loss of minerals from hard dental tissues, resulting from the destruction of HAp by acids. The initial attack creates a concentration gradient that promotes the dissolution of calcium and phosphates, leading to direct loss of these minerals from the surface layers of hard dental tissues and an increase in intercrystalline spaces within the enamel rods [15,16]. This facilitates the movement of acids and mineral ions into and out of the already porous structure. This, in turn, further facilitates acid diffusion due to lower crystal density [15,16] (Figure 2).

Therefore, demineralization involves active mineral loss in the advancing front of the lesion, transport of acids from the saliva and/or plaque toward the advancing front, and transport of mineral ions in the opposite direction [17,18]. Thus, over time, the advancing front of the lesion deepens into the hard dental tissues, while the relatively intact surface layer above the lesion modulates the reaction between the internal structure and the external solution [17,18].

Although previously the demineralization process has been described as a diffusion reaction, determined by the rate of transport of mineral ions to and from the advancing front of the lesion, recent evidence suggests it has a surface-controlled demineralization mechanism, where the reaction rate is determined by chemical dissolution processes at the crystal level of the advancing front of the developing lesion [19]. However, it is likely that dissolution occurs by a combination of these two mechanisms [20]. The porosity of solid dental structures, the presence of dissolution inhibitors in saliva, mineral solubility, and the size of the exposed surface for dissolution may influence the speed and extent of dissolution processes [19,20].

Demineralization occurs when weak organic acids attack dental tissues, releasing mineral components. The process causes mineral ions to be released into solution:

$$Ca_{10}(PO_{4}{)}_{6}(OH{)}_{2}+14H^{+}\to 10Ca^{2+}+6H_{2}PO_{4}^{-}+2H_{2}O$$

The rate of demineralization is inversely proportional to the degree of saturation of calcium and phosphate ions and the pH value of the solution [21,22].

In the early stages of demineralization, the tooth surface remains intact, and demineralization is reversible. However, if acidic conditions persist below the critical pH value of about 5.5, acid-induced dissolution leads to a gradual loss of tooth minerals, resulting in irreversible changes and cavity formation [23,24].

Remineralization is defined as a process in which calcium and phosphate ions supplied from an external source (not from the tooth itself) are deposited in the empty spaces in the crystal lattice of the demineralized dental tissues [25,26]. This represents a natural mechanism for returning the minerals to a crystalline form, i.e., into the HAp crystal lattice. It occurs under almost neutral, physiological pH conditions, where calcium and phosphate mineral ions from saliva and dental plaque fluid are redeposited into the demineralized lesion, resulting in the formation of HAp crystals that are larger and more resistant to acid dissolution than the original HAp crystals [25,26].

Demineralization and remineralization cycles constantly move between net loss and net gain of minerals. When the balance tends toward net loss over a longer time interval, clinical signs of the demineralization process can be recognized. The long-term outcome of this cyclical process depends on multiple internal and external factors and involves interactions between the environment, patient behavior, and patient genetics [27,28] (Table 1).

## 4. Dental Plaque and Dental Caries

Dental caries is not just a continuous and unidirectional process of mineral phase demineralization, but a cyclical process with periods of demineralization and remineralization; it occurs when demineralization exceeds remineralization [29,30].

Modern dentistry aims to prevent caries progression and deal with non-cavitated lesions in a non-invasive or minimally invasive manner (Table 2). The progression of tooth caries is a process in which, if acted upon in its early stages with non-invasive intervention, it can lead to the conversion of an active lesion into an inactive one [31,32].

The occurrence of dental caries is due to cariogenic bacteria (mutans streptococci and lactobacilli) from dental plaque, which, in the presence of fermentable carbohydrates (e.g., glucose, fructose, starch), produce organic acids (e.g., lactic, acetic), causing a drop in pH value at the tooth surface [33,34].

There is a constant steady state between solid HAp in enamel and dissolved components of HAp in plaque biofilm. However, according to new knowledge, the mixed bacterial-ecological theory is increasingly advocated, in which the previously mentioned cariogenic bacteria are only part of the potentially cariogenic bacteria present in plaque [35,36]. It is believed that multiple bacterial species have the potential to cause carious lesions to develop, depending on dietary factors and the acidogenicity of commensal oral bacteria, leading to ecological changes in the plaque bacterial community and, consequently, increased caries risk [35]. Repeated consumption of easily fermentable carbohydrates, especially sucrose, leads to a proportional increase in the overgrowth of cariogenic bacteria such as *S. mutans*. These biofilm changes increase the potential for enamel mineral loss, organic acid production, and changes in oral microflora, leading to an increased risk of carious lesion development [35,36].

Dental caries is currently understood and defined in cariology as a “biofilm-mediated, sugar-driven, multifactorial, dynamic disease that results in the phasic demineralization and remineralization of dental hard tissues” [37].

When oral cavity colonization by cariogenic bacteria occurs, the balance of factors favors demineralization. To prevent this, changes are needed to prevent demineralization and direct the dissolution process in favor of remineralization, i.e., to form solid dental tissue [38,39].

## 5. *Macula alba*

The earliest macroscopic clinical evidence of enamel caries is the presence of a “white lesion,” “white spot,” or “*macula alba*”. The chalky-white color of the white spots, compared to sound enamel, results from increased porosity, which changes the light diffraction properties of the enamel [40,41].

**Figure 3:**
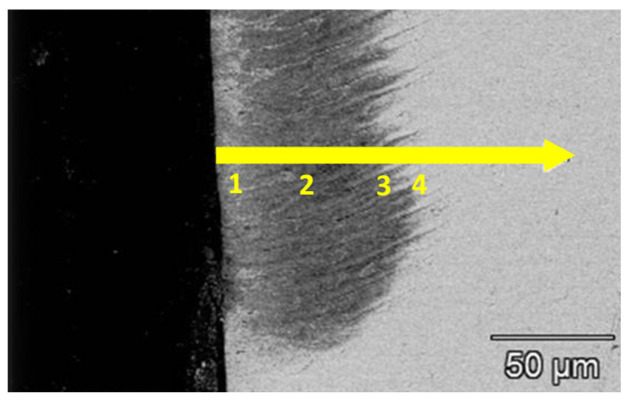
*Macula alba* on a longitudinal section: (1) superficial, relatively intact but slightly porous zone; (2) demineralized lesion body; (3) dark zone—an area of remineralization; (4) translucent zone, representing the advancing front of the lesion (arrow from the surface toward the deeper layer of enamel).

White spot lesions (WSLs) are characterized by subsurface demineralization under an apparently intact, mineralized surface layer. The histological appearance of WSL manifests as a rough geometric profile consisting of a relatively unchanged surface layer with increased porosity in the most superficial aspect of the lesion, covering the demineralized lesion body, which constitutes the largest part of the carious lesion where the greatest mineral loss occurs (Figure 3). Deep below the demineralized lesion is unchanged, sound enamel [42,43].

The surface zone is essential for controlling the rate and degree of demineralization and remineralization processes. Its collapse and destruction lead to cavitation, which is why, in clinical conditions, probing these lesions should not be performed [44,45].

Although at first glance the reintroduction of minerals from saliva into porous demineralized enamel seems simple, it is actually a complicated process. One challenge relates to limiting remineralization by ion diffusion from the external solution [46,47]. With excessive fluoride concentrations and/or high enamel supersaturation conditions, minerals can quickly deposit on the enamel surface layer, filling surface enamel porosities and disrupting the connection between the external environment (saliva and plaque fluid) and the internal environment (subsurface layer, i.e., lesion body). Furthermore, mineral deposition at a specific depth within the carious lesion depends on the local availability of partially demineralized crystals, which act as mineral scaffolds, as well as local supersaturation, fluoride levels, and pH value. However, additional factors such as the presence of salivary proteins play a vital role [46,47].

A WSL that ceases to progress and undergoes natural surface hardening through remineralization is referred to as an arrested or inactive lesion. In line with minimally invasive dentistry principles, non-cavitated WSLs should not be surgically restored; remineralization and preventive strategies are the appropriate management. Unlike active lesions, inactive lesions are inherently stable and respond poorly to remineralization therapies. Therefore, clinical lesion activity is vital for determining susceptibility to remineralization therapy and preventing overtreatment of lesions that are chemically stable [48,49].

## 6. Role of Saliva in the Remineralization Process

The key to modern caries therapy is the assessment of caries risk and the selection of appropriate remineralizing therapies for patients who need them [50,51].

Saliva is critical for determining caries risk, but also a critical biological protective factor in the enamel remineralization process [50]. Greater buffering capacity and salivary secretion rate reduce the rate and degree of demineralization [50]. Saliva can neutralize acids, form a protective membrane on tooth surfaces, and promote remineralization by supplying calcium, phosphate, and fluoride to enamel and dentin. Saliva pH directly affects remineralization by determining the amount of calcium and phosphate ions available to enamel through saliva during an acid challenge [51]. Saliva can inhibit tooth demineralization during periods of low pH value, while simultaneously promoting tooth remineralization after the pH value returns to neutral [50,51].

Systemic diseases, hereditary disorders, various medications, and other medical interventions may have detrimental effects on saliva production, buffering capacity, and the amount of calcium and phosphate available for remineralization [52]. Saliva’s protective properties also depend on its quantity. These protective properties can be significantly strengthened or weakened based on secretion rate under unstimulated and stimulated conditions. Unstimulated, normal saliva secretion is greater than 0.3 mL/min, ranging between 0.5 and 1.5 L per day. Individuals with reduced saliva flow have more acidic saliva and biofilm, increasing the risk of additional demineralization [52]. Reduced saliva flow also creates an oral environment unable to effectively neutralize acids, resulting in a prolonged decrease in intraoral pH value [53]. The remineralization process is often impaired in patients with reduced saliva production, and fluoride use may be limited due to a lack of calcium and phosphate ions [52,53].

Normal saliva is supersaturated with calcium and phosphate ions that help prevent HAp crystal decomposition. As main components of HAp crystals, calcium and phosphate concentrations in saliva and plaque play a key role in tooth demineralization and remineralization processes [54,55]. At equal saturation degree, optimal enamel remineralization rate can be achieved with a calcium/phosphate ratio of 1.6. In plaque fluid, the calcium/phosphate ratio is approximately 0.3, so additional calcium supply can increase enamel remineralization [54,55].

## 7. Remineralization Agents

In recent years, dentistry’s focus has been directed toward a conservative approach, in which remineralization procedures are an optimal way to regenerate lost tooth structure [56,57]. The preventive approach of identifying, conserving, and non-restorative treatment of initial caries saves both the dental workforce and costs, as well as patient discomfort [56,57].

Initially, these processes relied solely on formulations of various fluoride types, which restored HAp crystals by supplying them with necessary ions that were partially lost from the crystal lattice [58]. Later, new products for biomimetic remineralization were successfully introduced, having the ability to create apatite crystals in completely demineralized collagen fibers [58,59].

In order to manage caries lesion development by minimizing enamel solubility during acid attack, individual tooth surfaces should be exposed to supersaturated levels of calcium, phosphate, and fluoride that are available in products containing these ions in bioavailable form [60,61].

The use of fluoride toothpastes has led to significant caries reduction at the population level. However, fluoride remineralizing efficacy depends on simultaneous bioavailability of calcium and phosphate ions; when these are depleted during intense acid challenge, fluoride alone cannot drive full remineralization [62,63]. In this context, it is possible to use plaque as a reservoir for calcium and phosphate-based agents. Plaque fluid is supersaturated with calcium phosphates at a neutral pH value. However, at cariogenic pH values, lesions are porous, and there is a possibility of mineral penetration and accelerated fluoride-hydroxyapatite deposition [62,63].

Numerous remineralizing agents and remineralization techniques have been investigated, and many are used clinically, with significant positive results [64,65]. The proposed classification in Table 3 is organized by primary mechanism of action. Fluoride-based agents act by promoting fluorapatite formation and inhibiting bacterial metabolism. Calcium-phosphate systems replenish ionic bioavailability to drive mineral deposition. Nanotechnology-based systems exploit nanoparticle morphology for enhanced surface interaction and controlled ion release. Biofilm modifiers address the microbial ecology of caries without necessarily remineralizing enamel directly. Biomimetic and emerging systems replicate biological mineralization processes at the molecular level. Adjunctive antimicrobial therapies target the cariogenic biofilm, but do not remineralize enamel per se and are therefore listed separately from the remineralizing categories above.

### 7.1. Fluorides in Remineralization

The presence of fluoride during de/remineralization cycles leads to its incorporation into the HAp crystal structure, and thereby, not only is crystal solubility reduced, but the rate of calcium and phosphate deposition in enamel is increased due to the lower solubility of fluorapatite (FAp). Fluoride reduces enamel solubility because the fluoride ion is more stable than the hydroxide ion and firmly binds with calcium ions in the crystal lattice [66,67] (Table 4).

With each intake of sugar, the pH value of dental plaque decreases due to acid production. This leads to enamel dissolution. However, if fluorides are present in plaque fluid and the pH value is not lower than the critical pH value, HAp dissolves and FAp forms simultaneously. Furthermore, FAp deposits on the enamel surface layer, while HAp dissolves from the subsurface [68,69].

The leading role of fluorides in preventive dentistry is indisputable [70,71]. However, fluoride effectiveness in enamel remineralization may be limited by the bioavailability of calcium and phosphate ions [72,73]. If the acid challenge to enamel is extensive, the salivary reservoir of calcium and phosphates is quickly depleted, and net mineral loss from enamel can occur. Increased calcium and phosphate concentrations increase fluoride retention in plaque [74,75]. Therefore, for remineralization to occur in patients with increased caries risk, increasing bioavailable calcium and phosphates is fundamentally important for improving the effectiveness of remineralization agents [76,77].

### 7.2. New Remineralizing Agents, Biomimetic Remineralization of Dentin and Enamel

Relatively new remineralization research focuses on biomimetic remineralization materials that can form apatite crystals within completely demineralized collagen fibers. Biomimetic remineralization aims to fill demineralized collagen with amorphous calcium phosphate (ACP) particles, which is the initial, solid phase, and a transient phase in biomineralization, deposited as a HAp precursor [77]. These prenucleation clusters (≈1 nm in diameter) aggregate into larger (10–50 nm in diameter) ACP nanoparticles. They then penetrate into the intrafibrillar spaces of collagen fibrils and form a metastable crystal phase. These crystals eventually merge into individual apatite crystallites in the zone between collagen molecules [77].

Several biomimetic agents are used for the remineralization of hard dental tissues (Table 3). However, they should also meet the requirements listed in Table 5 [78,79]. These different remineralization agents are considered in the following sections.

#### 7.2.1. Amorphous Calcium Phosphate (ACP)

ACP has the ability to rapidly provide calcium and phosphates, and it possesses high bioavailability, even in patients with low salivary flow. Additionally, ACP is able to bind with fluorides, forming a new FAp layer on the tooth [81,82].

To prevent calcium and phosphorus components from reacting with each other before use, ACP technology requires a two-phase delivery system, where calcium and phosphorus salts are kept separate until used. Upon mixing, they rapidly form ACP, which can deposit on the tooth surface. This deposited ACP can then easily dissolve in saliva and thus be available for tooth remineralization [83,84].

High concentrations of calcium and phosphate can exist at low pH values, but as pH values increase, calcium and phosphate precipitate [81,83,85]. Calcium and phosphate supersaturation in the immediate proximity to the tooth, at low pH values, helps prevent HAp crystal dissolution, while, as a result of the concentration gradient, as the pH value increases, the balance between demineralization and remineralization changes, and ACP and fluorides deposit onto the tooth surface [81,83,85]. ACP can then convert to HAp, which is less soluble than ACP. HAP deposited in dental structures as apatitic mineral is similar to HAp naturally found in teeth and bones [81,83,85]. This can help prevent demineralization because, at a given pH value, ACP is more soluble than hydroxyapatite crystals, so ACP will dissolve first and supersaturate the surface with calcium and phosphate ions, helping prevent HAp crystal dissolution [85,86].

ACP’s amorphous structure also allows fluorides and other ions to be incorporated into it and can increase fluoride bioavailability. Additionally, ACP offers both cosmetic and preventive effects [87]. If there are scratches and surface defects on the enamel surface, light disperses in such a way that the tooth may appear darker [88]. Unlike hydrogen peroxide and other chemical cleaning agents that remove staining spots, ACP filling of surface defects changes light dispersion, improving aesthetics [87,88].

#### 7.2.2. Tricalcium Phosphate (TCP)

Research shows that combining TCP with fluoride can provide better enamel remineralization and create acid-resistant minerals compared to using fluorides alone [89]. Formation of calcium-phosphate complexes (if fluoride is present, calcium fluoride forms) is a disadvantage that inhibits remineralization by reducing bioavailable calcium and fluoride levels [89]. Therefore, a protective organic coating around calcium is added, preventing unwanted interactions with fluoride, but it can dissolve when particles contact saliva [89]. During toothbrushing, TCP comes into contact with saliva, causing the barrier to dissolve and release calcium, phosphate, and fluoride [90]. TCP is also considered an agent that will enable increased calcium levels in plaque and saliva [89,90].

#### 7.2.3. Dicalcium Phosphate Dihydrate (DCPD)

DCPD is a calcium phosphate salt and recognized HAp precursor that converts to fluorapatite (FAp) in the presence of fluoride ions, making it a useful adjunct in fluoride-containing dentifrices [91]. Adding DCPD to toothpastes increases calcium incorporation into enamel and elevates free calcium ion levels in the plaque fluid, creating a more favorable ionic environment for remineralization [92]. While in vitro and in situ data are promising, further long-term RCTs are needed to confirm their superiority over standard fluoride formulations [91,92].

#### 7.2.4. Arginine Bicarbonate–Calcium Carbonate

Arginine bicarbonate (an amino acid complex) binds calcium carbonate particles (a common abrasive used in toothpastes) to enamel or dentin surfaces [93]. When calcium carbonate dissolves, the released calcium is available for solid dental substance remineralization, while carbonate release can provide a mild local pH increase [93]. Arginine significantly increases fluoride uptake compared to using fluorides alone, and initial lesions treated with toothpaste containing arginine bicarbonate also show better fluoride penetration compared to those treated with conventional fluoride toothpaste [93,94]. However, calcium carbonate’s poor solubility is a disadvantage because saliva calcium levels hardly change [93,94].

#### 7.2.5. Recaldent™ (Casein Phosphopeptide–Amorphous Calcium Phosphate (CPP-ACP))

**Figure 4:**
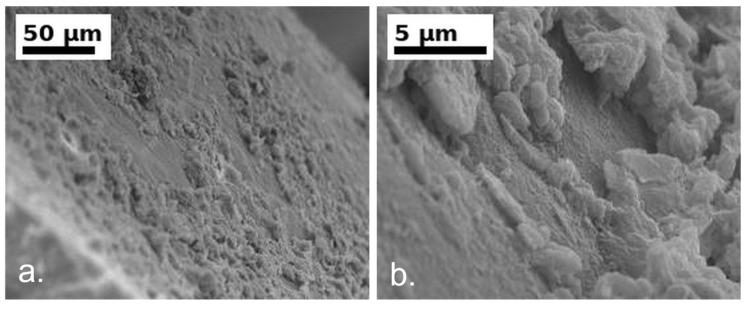
(**a**,**b**) Amorphous deposits on the enamel surface after application of toothpaste with CPP-ACP.

The use of casein phosphopeptides (CPPs) as anticariogenic and anticalculus agents began with Reynolds in the 1990s [95]. CPPs are multiphosphorylated peptides present in milk and are obtained from casein through proteolytic degradation of the dairy products αS1-, αS2-, and β-casein [46]. CPPs containing the cluster sequence Ser(P)-Ser(P)-Ser-(P)-Glu-Glu stabilize ACP nanoclusters, leading to increased calcium phosphate levels in dental plaque [46,65]. CPPs have the ability to stabilize high concentrations of calcium and phosphate in metastable solution; i.e., CPP complexes bind and form clusters with calcium and phosphate, preventing crystal growth to the critical size needed for nucleation and crystallization while providing a ready source of ionic calcium and phosphate [46,65] (Figure 4).

CPP complexes with ACP have proven anticariogenic activity (Table 6). CPP’s ability to buffer free calcium and phosphate ions enables supersaturation relative to enamel, reducing demineralization and enhancing remineralization. Therefore, the ability to provide supersaturated levels of ionic calcium and phosphate on tooth surfaces increases the effectiveness of remineralization. Agents using Recaldent™ technology penetrate lesions below the surface through the porous enamel surface [96,97].

Upon lesion penetration, CPPs have a high affinity for apatite binding. With increasing pH value, bound ACP level increases, stabilizing free calcium and phosphate and thus providing anticalculus action [98].

Calcium phosphate in these complexes is biologically available for intestinal absorption, and this concept is applied to create agents with bioavailable calcium and phosphate in an appropriate form and molecular ratio for subsurface enamel lesion remineralization (pastes, creams, restorative materials, chewing gums, etc.) [99,100,101].

A disadvantage is that Recaldent™-containing agents should not be used in individuals sensitive to milk or milk proteins, and where allergic reaction risk exists.

Recent systematic reviews have evaluated CPP-ACP’s clinical efficacy. A systematic review of 14 randomized clinical trials confirmed CPP-ACP’s effectiveness in WSL remineralization, with greater efficacy versus placebo and resin infiltration [102]. Another meta-analysis found similar clinical efficacy between CPP-ACP and topical fluorides, suggesting CPP-ACP as a viable alternative, particularly for patients at risk of fluorosis [103]. The latest clinical trials demonstrate effectiveness for both in-office and home treatment protocols [104,105].

CPP-ACP containing fluorides provides remineralization superior to both CPP-ACP and conventional and high-fluoride toothpastes. Adding xylitol also increases remineralization potential [106,107].

#### 7.2.6. Bioactive Glasses (Calcium-Sodium-Phosphosilicate)

Bioactive glasses (BGs; most commonly used are Bioglass^®^45S5 and NovaMin^®^; Table 7) are a class of bioactive materials consisting of calcium, sodium, phosphate, and silicate. They are reactive when exposed to body fluids and deposit calcium phosphate on solid dental tissue surfaces [108,109].

Recent developments include strontium-doped formulations and modified glass compositions with controlled ion release, where strontium-doped bioactive glass achieved superior mineral regain compared to NovaMin and BioMin [110]. However, previous systematic reviews noted limited clinical evidence for NovaMin remineralization versus fluoride toothpastes and a necessity for rigorous clinical validation [111,112]. Recent in vitro studies show promise for bioactive glass formulations in remineralization and antibacterial activity [113,114].

BG particles in aqueous environments immediately begin surface reactions, and these occur in three phases: (1) cation leaching and exchange (when BG contacts saliva, it rapidly releases sodium, calcium, and phosphorus ions into saliva, and these are available for tooth surface remineralization); (2) SiO_2_ network dissolution; (3) calcium and phosphate precipitation and apatite layer formation [112]. Within 3–6 h, the calcium phosphate layer will crystallize into hydroxy–carbonate–apatite (HCA), which is essentially the binding layer [112].

**Figure 5:**
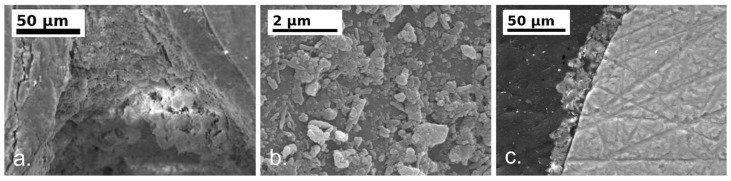
(**a**) Formation of a bioactive interfacial layer resembling an ion-exchange layer in glass-ionomer cements; (**b**) angular deposits on the enamel surface after application of BG paste; (**c**) creation of a layer of “artificial enamel” as a protective layer over the enamel.

Chemically and structurally, this apatite is almost identical to bone and tooth mineral [108,109]. BG surface reactions from initial reaction to HCA layer formation of 100–150 μm take 12–24 h, but these particles release ions and transform into HCA for up to 2 weeks [108,112]. Eventually, these particles will completely transform into HCA and create a mineralized layer that is mechanically strong and acid-resistant. Continuous calcium release over time maintains dentin protective effects [112,113] (Figure 5).

BG leads to increased fluoride bioavailability and increased fluoride uptake by solid dental substances. Besides being an anticariogenic agent, BG can be used in the treatment of hypersensitivity and for tooth whitening [115,116,117].

#### 7.2.7. Nanoparticles

Nanoparticles more easily release ions compared to microparticles and are often added to restorative materials as inorganic fillers and fluoride ions for hard dental tissue remineralization [118]. Calcium fluoride nanoparticles increase cumulative fluoride release compared to fluoride release from traditional glass-ionomer cements because nanoparticle CaF_2_ (nano-CaF_2_) has 20 times larger surface area compared to microparticles [118]. Calcium-phosphate-based nanomaterials use HAp, TCP, and ACP nanoparticles as sources of calcium/phosphate ion release and increase HAp supersaturation during initial carious lesion treatment [118,119].

##### Nano-HAps

**Figure 6:**
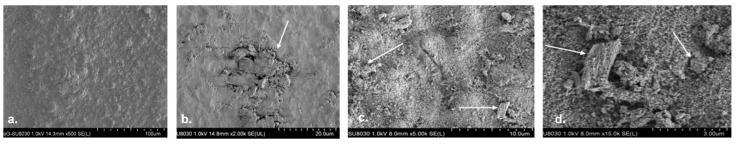
(**a**–**d**) Deposits of HAp particles (arrows) after application of HAp toothpaste on the enamel surface.

Nanometric-sized HAp particles (nano-HAps), according to morphology and crystal structure, are similar to apatite crystals in enamel, enabling biomimetic repair of enamel’s natural mineral component through creating a thick and homogeneous apatite layer. Additionally, nano-HAps are thought to inhibit oral biofilm formation [120] (Figure 6).

Nano-HAps have the potential to remineralize initial enamel lesions, with a 10% nano-HAp concentration potentially being optimal for early enamel caries remineralization [120]. HAp particles sized 20 nm fit well with the dimensions of enamel surface nanodefects and can firmly adhere to demineralized enamel surfaces and inhibit further acid attack. Commercially available pastes containing nano-HAp are effective in reducing dentin hypersensitivity [120,121].

Recent clinical evidence has strengthened support for nano-HAp as an effective remineralizing agent [122]. A randomized clinical trial demonstrated that nano-hydroxyapatite gel with ozone therapy achieved 69.3% remineralization at one-year follow-up for approximal initial caries [123]. A pediatric randomized clinical trial showed significant enamel remineralization with a zinc carbonate hydroxyapatite-based toothpaste after one month of treatment [124]. Several systematic reviews have confirmed nano-HAp’s efficacy comparable to fluoride treatments, with additional benefits for managing hypersensitivity and molar–incisor hypomineralization in several in vitro and short-term clinical studies [122,125,126,127]. However, most supporting evidence derives from in vitro and short-term in situ studies; long-term RCT data remain limited, and head-to-head comparisons with fluoride under standardized conditions are still needed [122,126,127].

#### 7.2.8. Herbal Preparations

Active plant extracts from the Labiatae family, juice from the *Vaccinium* plant, and compounds from the essential oil of *Coleus forskohlii* show significant inhibitory action against *S. mutans* [128,129].

Theobromine is a member of the xanthine family found in cocoa (240 mg/cup) and chocolate (1.89%) [130] and has demonstrated improvement in enamel crystal growth. It provides promising results in enamel remineralization and microhardness [131].

Grape seed extract is a rich source of naturally derived polyphenolic compounds, including proanthocyanidins. These plant polyphenols have demonstrated the ability to promote calcium fluoride formation on enamel surfaces, suggesting a potential role in enhancing enamel remineralization. Their interaction with calcium and fluoride ions at the enamel surface may contribute to the formation of a protective mineral layer, supporting caries prevention [132].

Polydopamine is a bioinspired polymer produced by the oxidative self-polymerization of dopamine, mimicking the adhesive proteins found in marine mussels. It forms a thin, adherent coating on a wide variety of surfaces, including enamel, and has been shown to promote fluoride-mediated enamel remineralization by enhancing mineral deposition on demineralized surfaces [133].

The evidence for the herbal preparations studied in enamel remineralization is mainly based on in vitro and animal studies; relevant clinical studies should be conducted in the future to ascertain their appropriateness.

#### 7.2.9. Probiotics

Probiotics are living microorganisms that, according to the WHO, when administered in appropriate quantities, have positive effects on the host’s health [134,135]. They act either by disrupting dental plaque or by producing antimicrobial compounds that inhibit oral bacteria [136,137]. Long-term consumption of milk containing *L. rhamnosus* GG strain reduces the progression of initial caries [138]. The use of *L. reuteri* ATCC 55739 and *Bifidobacterium* DN-173 010 reduces the amount of *S. mutans* in saliva [139,140]. Certain strains of *Lactobacillus*, including *L. reuteri* CF2-7F (ATCC PTA-4965), MF2-3 (ATCC PTA-4964), FJProdentis (ATCC PTA-5289), and FJ3 (ATCC PTA5290), have good antimicrobial action against *S. mutans* [141,142]. Up-to-date, most evidence derives from short-term interventions, rather than clinical caries incidence.

## 8. Adjunctive Therapies

While remineralizing agents directly target mineral restoration of dental hard tissues, a comprehensive caries management strategy also requires approaches that address the underlying microbial etiology of the disease. Adjunctive therapies do not remineralize enamel per se but act by modifying the cariogenic biofilm, eliminating or replacing pathogenic bacteria, or disrupting the ecological conditions that favor demineralization. These therapies include substitution therapy, bacteriophage therapy, sugar substitutes, photodynamic therapy, and laser-aided mineralization and are considered complementary components of modern, minimally invasive caries prevention protocols.

### 8.1. Substitution Therapy

Substitution (replacement) therapy emerges with advances in genetic engineering and recombinant DNA technology, which affects pathogens in the oral microflora, where mutated strains of *S. mutans* lack the ability to metabolize carbohydrates into acids [143,144]. For example, the BCS3-L1 strain of *S. mutans* has been developed, which does not produce acid but is active against natural cariogenic strains of *S. mutans* with the aim of replacing them in the oral cavity [145]. This type is less cariogenic and more stable [146]. Another type of mutation, through deletion of the GTF-C gene, blocks the ability of *S. mutans* to produce extracellular glucans [147,148].

### 8.2. Bacteriophage Therapy

Bacteriophages are viruses that attack bacteria. Their characteristics include target specificity: they are directed toward patients with antibiotic allergy, are cost-effective, and are generally well tolerated [149,150]. There are bacteriophages that lyse *S. mutans* [151,152].

### 8.3. Sugar Substitutes

Sugar substitutes are alternative sweeteners and can be artificial or natural. Stevia is a sugar substitute from the Asteraceae family that, in addition to systemic effects, also has anticariogenic characteristics, reduces biofilm formation, and has positive effects at the periodontal level [153,154]. It is active against *S. mutans*, *S. sobrinus*, *L. acidophilus*, and *C. albicans* [153,154].

Xylitol is a non-fermentable sugar alcohol that inactivates *S. mutans* and inhibits the plaque’s ability to produce acids and polysaccharides [155,156]. When consumed as lozenges or chewing gum, it stimulates increased flow of alkaline saliva rich in minerals from the minor salivary glands, resulting in increased buffering capacity and faster remineralization of damaged enamel [155,156].

### 8.4. Photodynamic Therapy (PDT)

PDT is a treatment that uses light to activate a photosensitizing agent in the presence of oxygen, resulting in the formation of a reactive radical that causes cell death and has promising results in the inactivation of microorganisms associated with caries [157,158]. For PDT against *S. mutans*, erythrosin is a suitable photosensitizing agent because it acts effectively against Gram-positive bacteria like *S. mutans* [159,160]. This requires a light source with a wavelength of approximately 530 nm, which can also be achieved with LED diodes [161].

### 8.5. Laser-Aided Mineralization

Because of their photothermal properties, lasers can assist in crystal growth by warming the surrounding area and changing reaction conditions to simulate a hydrothermal environment. Additionally, femtosecond pulsed lasers (fs) can be used to remineralize enamel by sintering artificial fluorapatite powder placed on the surface of demineralized enamel, which contains iron oxide nanoparticles in chitosan. Like a thermal antenna, these nanoparticles can absorb laser photons, disperse the heat locally to fluorapatite crystals, and cause densification into a dense layer that adheres to the original enamel [162].

## 9. Clinical Application of Remineralizing Agents

The clinical application of remineralizing agents depends on the product formulation, indication, and patient risk profile. Table 8 summarizes the main agents, their available formulations, methods of application, and recommended frequency of use.

## 10. Comprehensive Prevention Protocols

Effective caries management requires an integrated, multifaceted approach that combines fluorides with calcium-phosphate-based remineralizing agents and antimicrobial strategies [11,80,102,103]. The evidence presented in this review demonstrates that modern remineralization protocols should be tailored to individual patient risk profiles, combining multiple agents to maximize therapeutic benefit.

For patients at high caries risk, combination therapy using fluoride-containing products supplemented with CPP-ACP or nano-hydroxyapatite offers synergistic remineralization effects superior to fluoride monotherapy [102,103,122,124]. Recent clinical trials confirm that BG formulations and calcium phosphate compounds can effectively supplement fluoride therapy, particularly in patients with reduced salivary flow or those at risk of fluorosis [110,112,113].

The selection of remineralizing agents should be guided by patient-specific factors. For pediatric patients and individuals concerned about fluoride exposure, nano-hydroxyapatite and CPP-ACP represent effective non-fluoride alternatives with efficacy comparable to that of fluoride treatments [99,100,101,102]. For patients with dentin hypersensitivity, bioactive glass formulations and nano-hydroxyapatite provide dual benefits of remineralization and tubule occlusion [113,116,120,121]. In cases of active WSLs, combination protocols incorporating both in-office professional applications and home-use products may offer superior outcomes compared to single-agent approaches [11,105].

Emerging evidence highlights that bioavailable calcium, rather than fluoride concentration alone, may be the critical limiting factor in remineralization processes [80,100,163,164]. This paradigm shift emphasizes the importance of calcium and phosphate delivery systems in modern caries prevention. Contemporary protocols need not rely exclusively on high fluoride concentrations; instead, optimal remineralization can be achieved through balanced formulations that ensure adequate bioavailability of calcium, phosphate, and fluoride ions at the tooth surface [11,165,166].

## 11. Current Challenges and Future Perspectives

Despite substantial advances, key challenges persist across the field. A major limitation is the lack of standardized outcome measures, which impedes direct comparisons between agents. Most evidence for nano-HAps, bioactive glass, and herbal preparations derives from in vitro or short-term in situ models. Long-term RCTs with harmonized caries activity outcomes remain scarce for several agents, and translation to routine clinical practice has been slow.

Priority areas for future research include the following: (1) personalized protocols guided by caries risk profiling and salivary diagnostics; (2) nanotechnology-based delivery systems with enhanced lesion penetration; (3) clinical validation of biomimetic peptide scaffolds; and (4) multi-agent strategies targeting both ionic (calcium, phosphate, fluoride) and biological (biofilm, pH) dimensions of caries risk simultaneously. REFIX technology, a novel approach based on multifunctional phosphate-based dental gel technology in an acidified stabilized phosphate/fluoride complex, which is established especially in saliva [167], has shown early promise in in vitro enamel remineralization studies and warrants further clinical investigation.

## 12. Conclusions

Among agents with the strongest clinical evidence, fluoride remains the cornerstone of caries prevention. CPP-ACP and nano-hydroxyapatite have demonstrated comparable remineralizing efficacy to fluoride in multiple systematic reviews and RCTs, with particular utility in fluorosis-risk and pediatric populations. Bioactive glass formulations show strong in vitro and growing clinical evidence, especially for dentin hypersensitivity and patients with reduced salivary flow.

Bioavailable calcium, rather than fluoride concentration alone, is the critical rate-limiting factor in remineralization under salivary depletion conditions. Combination protocols ensuring simultaneous delivery of calcium, phosphate, and fluoride appear to be superior to single-agent approaches, and agent selection should be individualized based on caries risk, salivary status, patient age, and lesion activity.

Experimental approaches, including bacteriophage therapy, substitution therapy, and laser-assisted mineralization, show promise but currently lack sufficient clinical evidence for routine recommendation. Standardization of outcome measures and long-term clinical trials are the most pressing research priorities.

Clinicians are encouraged to implement evidence-based, minimally invasive remineralization strategies that arrest non-cavitated lesions and preserve natural tooth structure.

## Figures and Tables

**Table 1:** Factors influencing de/remineralization cycles (modified from Abou Neel et al. [3]).

Biological Factors	Chemical Factors	Health and Education	Behavioral Factors
Saliva flow and quality	pH value	Current health status (general diseases, individual immune response, bulimia, gastro-esophageal reflux, etc.)	Dietary habits (frequency and timing of sugar consumption), fluoride exposure
Soft tissue anatomy	Acid type	Socio-economic status (plaque composition and amount, diet type)	Method and frequency of drinking different types of beverages (carbonated, fruit, etc.)
Dental tissue anatomy	Chelation potential	Medications (e.g., medications that lead to xerostomia, reduce saliva flow and pH value, and have a buffering effect towards acids)	Brushing frequency

**Table 2:** “Extension for prevention” has given way to new paradigms in minimally invasive dentistry. The minimally invasive approach incorporates detection, diagnosis, interception, and treatment of caries at the microscopic level.

Principles of minimal intervention in the management of dental caries (adopted at the FDI General Assembly, 2002, Vienna) [31]
Modification of the oral flora
Patient education
Remineralization of non-cavitated lesions of enamel and dentin
Minimal intervention of cavitated lesions
Repair of defective restorations

**Table 3:** Proposed classification of remineralizing agents. (Classification criteria and key references for each category are indicated in the text of Section 7. Abbreviations: TCP, tricalcium phosphate; ACP, amorphous calcium phosphate; CPP-ACP, casein phosphopeptide–ACP; HAp, hydroxyapatite; PDT, photodynamic therapy; AMPs, antimicrobial peptides.)

Fluoride-Based Agents		
**Fluoride boosters**	**Calcium-phosphate systems**	α- and β-tricalcium phosphate (TCP) Amorphous calcium phosphate (ACP) Casein phosphopeptide–amorphous calcium phosphate (CPP-ACP) Sodium calcium phosphosilicate (Bioglass) Dicalcium phosphate dihydrate (DCP)
	**Nanotechnology-based remineralization systems**	Calcium fluoride nanoparticles Calcium-phosphate-based nanomaterials Nano-HAp particles ACP nanoparticles Nano-bioactive glass materials
	**Non-calcium systems**	Arginine
**Biofilm modifiers**	Herbal preparations (theobromine, etc.) Probiotics Xylitol and other sugar substitutes Chemoprophylactic agents (triclosan, chlorhexidine, etc.) Polydopamine	
**Biomimetic and emerging remineralization systems**	Polyacrylic acid (PA) Oligopeptides Antimicrobial peptides (AMP) Biomimetic peptide scaffolding (self-assembling peptides-e.g., P11-4, amelogenin-derived peptides) Trimetaphosphate ion Chicken eggshell solution Keratin-based scaffolds	
**Adjunctive antimicrobial therapies (non-remineralizing)**	Bacteriophage therapy Replacement therapy Photodynamic therapy (PDT) High-intensity focused ultrasound Laser-assisted mineralization Electric field-induced remineralization	

**Table 4:** Mechanisms by which fluorides increase caries resistance.

Inhibition of demineralization and promotion of remineralization of incipient caries (accelerates the growth of new FAp crystals by reacting with calcium and phosphate ions)
Increased rate of maturation
Increased enamel resistance (FAp crystals are more resistant to acid attack compared to HAp crystals)
Interaction with microorganisms (inhibition of the activity of cariogenic bacteria by preventing the production of phosphoenol pyruvate (PEP), which is a key intermediate in the glycolytic pathway in bacteria)
Improved tooth morphology

**Table 5:** Characteristics of an ideal remineralizing agent (adapted from Zero [80]).

Has an advantage over fluorides
Diffuses and deposits calcium and phosphate in the subsurface layers
Does not deliver excess calcium ions
Does not favor calculus formation
Works at acidic pH
Works in patients with xerostomia
Enhances the remineralizing properties of saliva

**Table 6:** Mechanism of action of CPP-ACP.

Binding to the adhesion molecules of mutans streptococci, disrupting their incorporation into plaque.
Increasing the level of calcium ions in plaque, thereby inhibiting fermentation in it.
Providing protein and phosphate buffers to the pH value of plaque fluid, to suppress the excessive growth of acidogenic species of bacteria in the presence of excess fermentable carbohydrates.

**Table 7:** Composition of NovaMin™ bioactive glass.

Composition	Percentages
SiO_2_	40–60%
CaO	10–30%
Na_2_O	10–35%
P_2_O_5_	2–8%
CaF_2_	0–25%
B_2_O_3_	0–10%

**Table 8:** Clinical formulations, application methods, and frequency of use for the main remineralizing agents.

Agent	Formulations	Method of Application	Frequency/Indication
Fluoride	Toothpaste, varnish, gel, mouthrinse, foam	Brush-on (home); professional in-office (varnish/gel)	2× daily (toothpaste); 2–4×/year (varnish). Universal caries prevention [70,71]
CPP-ACP (Recaldent™)	Mousse, paste, chewing gum, varnish	Topical application; in-office or home use	1–2× daily (home); WSL, post-orthodontic, high caries risk [102,103]
Nano-HAp	Toothpaste, gel, mouthrinse	Brush-on; home use	2× daily; pediatric, fluoride-averse, hypersensitivity [122,124,126]
Bioactive Glass (NovaMin^®^)	Toothpaste, gel, varnish	Brush-on; topical application	2× daily; hypersensitivity, xerostomia, fluorosis-risk [112,113,116]
TCP	Toothpaste, mouthrinse	Brush-on; home use	2× daily; adjunct to fluoride, high caries risk [89,90]
Xylitol	Chewing gum, lozenges, toothpaste	Oral consumption (gum/lozenge); brush-on	3–5× daily (gum); biofilm modification, salivary stimulation [155,156]

## Data Availability

No new data were created or analyzed in this study. Data sharing is not applicable to this article.
